# Assessment of bacterial endosymbionts and the host, *Bemisia tabaci* (Hemiptera: Aleyrodidae), using rRNA and mitochondrial cytochrome oxidase I gene sequences

**DOI:** 10.1080/19420889.2018.1433442

**Published:** 2018-02-12

**Authors:** Tahseen Raza Hashmi, Salam Rita Devi, Naresh M. Meshram, Ram Prasad

**Affiliations:** a Division of Entomology, Indian Agricultural Research Institute, New Delhi, India; b Amity Institute of Microbial Technology, Amity University, Uttar Pradesh, India

**Keywords:** *Bemisia tabaci*, host plants, endosymbionts, distribution, genetic groups

## Abstract

Endosymbionts are vital factor for arthropod ecology. The whitefly *Bemisia tabaci* (Hemiptera: Aleyrodidae) is a cryptic species complex composed of more than 34 putative species. Moreover to the primary endosymbiont *Portiera aleyrodidarum*, six secondary endosymbionts *Cardinium*, *Arsenophonus*, *Rickettsia*, *Wolbachia, Hamiltonella* and *Fritschea* are known in *B*. *tabaci*. Here, we tested four of the six secondary endosymbiont lineages (excluding *Fritschea* and *Hamiltonella*) from 180 whitely individuals collected from six host plants belonging to families Solanaceae (Brinjal, Tomato and Potato) and Fabaceae (Soyabean, Mungbean and Subabool). Phylogenetic studies grounded on the mitochondrial cytochrome I gene revealed the presence of Asia 1, Asia II 1 and Asia II 7 genetic groups for *B. tabaci*. Specific primers targeting 16S rRNA and 23S rRNA gene were used for estimating the bacterial endosymbionts. As a primary endosymbiont *Portiera aleyrodidarum* was present in all the studied samples; whereas, an uneven distribution of secondary endosymbionts were recorded. Overall our finding exposes the variation and diversity of endosymbionts within the *B. tabaci* collected from different host plants and outlines the genetic groups of the insect pest. The study delivers a significant information concerning the circulation of secondary endosymbionts with host preferences of *B. tabaci* and provides suggestion for progressive studies on targeting the specific endosymbionts with respect to host for the control measures.

## Introduction

The whitefly *Bemisia tabaci* (Hemiptera: Aleyrodoidea) is a wide-reaching pest and has triggered substantial destruction to legumes, flowers, vegetables, grains and cotton production through direct nourishing, emitting honeydew, bringing host plant phytotoxic disorders, and communicating more than 120 plant viruses. [1,2,3] The taxonomic state of the *B. tabaci* species complex is very debatable. Plentiful revisions on morphological, behavioral and genetic variation have recommended diverse images, such as biotypes and genetic groups, for unlike populations of *B. tabaci*. Though, [4] drawn-out the rank of genetic group variances in the *B. tabaci* complex to species level. The inferences of mating revisions, [5,6,7] backed the proposal for presence of species level variances. *Bemisia tabaci* are morphologically indefinite but express characteristic biological, physiological and genetic variation, and consequently, are well-thought-out as cryptic species complex. [4,8,9,10,11] The genetic group Mediterranean (MED) are extremely unaffected to numerous insecticides, while Middle East Asia Minor 1 (MEAM1) have very extraordinary fecundity [12,13,14]. Hence, these genetic groups are vastly invasive, i.e. they move local populations and establish themselves quickly in a new spot. Such invasive populations have been known from several parts of the biosphere. Consequently, accepting the population structure of *B. tabaci* is critical to govern its blowout and to prevent the types of destruction triggered by the different species. Within 34 putative species^11^ of *B. tabaci* defined globally, nine have been recognized from India and the distribution outlines of *B. tabaci* specify that extreme range is established in Southern and Eastern India. Miscellany drops nearby the north and north-west, where both Asia II-1 or Asia I genetic groups dominate. Remarkably from Delhi, which harbour Asia II-7 and some areas of Gujarat harbour MEAM1 has been described. The spreading outline of *B. tabaci* may be influenced by numeral elements composed with host plants, geographical location and frequent anthropogenic-derived actions related with trade [15].

Endosymbionts are prevalent in environment, mainly predominant in arthropods and are of two types primary endosymbionts and secondary endosymbionts [16]. Primary endosymbionts are known to exist in all host individuals, vertically communicated and deliver vital nutrients to the hosts. Conversely, the Secondary endosymbionts are facultative and both vertically and horizontally communicated [17,18,19]. Secondary endosymbionts can have abundant effects on host phenotype that upsurge their personal vertical diffusion rate to host offspring. They can upsurge the proportion of infested females in host populations by sexual manipulation [20] or can improve host fitness through behavioral or physiological modifications [21,22,23]. The notorious and considered secondary endosymbiont is *Wolbachia pipientis*, an *Alphaproteobacteria* predominant among various classes of arthropods and filarial nematodes [24]. In several insects, most bacteria identified are environmentally-inherited commensals or pathogens which reside the digestive tracts once they are used up by insects. Innumerable bacterial phyla are frequently present in insect guts, with ƴ-proteobacteria, α- proteobacteria, β-proteobacteria, *Bacteroidetes*, *Firmicutes* and others [25].

In whitefly *Portiera aleyrodidarum* is the only described primary endosymbionts, whereas a number of secondary endosymbionts, such as *Cardinium*, *Rickettsia*, *Arsenophonus*, *Wolbachia*, *Hamiltonella* and *Fritschea* are identified. Lately, one additional bacterium *Candidatus Hemipteriphilus asiaticus* in the native *B. tabaci* cryptic species China 1 has been anticipated. Phylogenetic examination stranded on the 16S rRNA and gltA genes offered that the bacterium goes to the *Alphaproteobacteria* subdivision of the *Proteobacteria* and has a close connection with human pathogens of the genus *Orientia* and named as *Orientia*-like organism (OLO) [26]. Endosymbionts are obligatory for the survival, blowout and expansion of the *B. tabaci* [27]. Bacterial variety in *B. tabaci* considered by countless investigators from diverse part of the world [26,28,29,30] but from India slight figures are available [31,32,33,34] on the dispersal occurrence of secondary endosymbionts of *B. tabaci*.

There are lot of studies available, which reveals the distribution of secondary endosymbionts with respect to their genetic groups and location but there is a lack of evidence on distribution frequency of endosymbionts with respect to the host preferences. Hence, the present study was aimed to define the comparative incidence of known endosymbionts of *B. tabaci* population from host plants belonging to family Solanaceae (brinjal, tomato and potato) and Fabaceae (soyabean, mungbean and subabool) collected from the fields of Indian Agricultural Research Institute, New Delhi, India. Additionally to finding the circulation frequency of endosymbionts with respect to host, the genetic groups of the collected *B. tabaci* were also determined.

## Results

### Occurrence of endosymbionts

**Figure 1.**
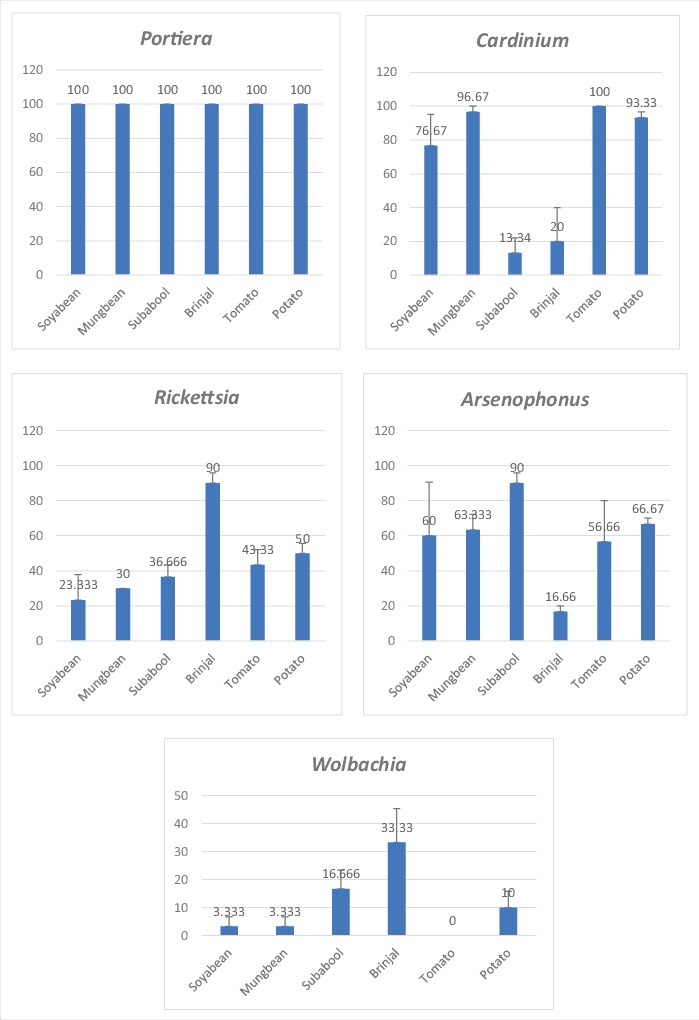
Distribution frequency of bacterial endosymbionts in *Bemisia tabaci* from Solanaceous and Malvaceous host plants.

Endosymbionts were recognized by using genus specific primers for the amplification of 16S rRNA gene of *Portiera, Rickettsia*, *Cardinium*, *Wolbachia*, and the 23S rRNA gene of *Arsenophonus* (Table 1). The occurrence of endosymbiotic bacteria, *viz*., *Portiera*, *Cardinium*, *Arsenophonus*, *Rickettsia* and *Wolbachia* in *B. tabaci* were perceived with diagnostic PCR in the samples collected from six host plants belonging to family Solanaceae (brinjal, tomato and potato) and Fabaceae (soyabean, mungbean and subabool). The incidence of primary endosymbiont, *Portiera* was 100% in all the samples, which stipulates the high quality of DNA extractions. [Fig. 1] explain the dispersal frequency of secondary endosymbionts in individual insects from each host. Individuals from certain hosts were found infected with all the described secondary endosymbionts unevenly*.*

**Table 1.** Primers used in PCR detection of endosymbionts and genetic groups.

Targeted gene	Primer's Sequence (5′→3′)	Annealing temp. (0C)/ Product size (bp)	Reference
*Portiera*16S rRNA	F-CGCCCGCCGCGCCCCGCGCCCGTCCCGCCGCCCCCGCCCGR- CCGTCAATTCMTTTGAGTTT	60/550	^45^
*Cardinium*16S rRNA	F- GCGGTGTAAAATGAGCGTGR- ACCTMTTCTTAACTCAAGCCT	58/400	^46^
*Rickettsia*16S rRNA	F- GCTCAGAACGAACGCTATCR- GAAGGAAAGCATCTCTGC	60/900	^47^
*Wolbachia*16S rRNA	F- CGGGGGAAAAATTTATTGCTR- AGCTGTAATACAGAAAGTAAA	55/700	^48^
*Arsenophonus*23S rRNA	F- CGTTTGATGAATTCATAGTCAAAR- GGTCCTCCAGTTAGTGTTACCCAAC	60/600	^27^
*B. tabaci*MtCOI	F- TTGATTTTTTGGTCATCCAGAAGTR- TCCAATGCACTAATCTGCCATATTA	52/800	^35^

In the studied individuals, the incidence of *Portiera* was 100% though a wide-ranging distribution frequency of secondary endosymbionts was noticed. Incidence of *Cardinium* in family Solanaceae was 20% in brinjal, 100% in tomato and 93.33% in potato; whereas, in family Fabaceae it was 76.67% in soyabean, 96.67% in mungbean and 13.34% in subabool. Incidence of *Rickettsia* in family Solanaceae was 90% in brinjal, 43.33% in tomato and 50% in potato; whereas, in family Fabaceae it was 23.33% in soyabean, 30% in mungbean and 36.66% in subabool. Incidence of *Arsenophonus* in family Solanaceae was 16.66% in brinjal, 56.66% in tomato and 66.67% in potato; whereas, in family Fabaceae it was 60% in soyabean, 63.33% in mungbean and 90% in subabool. Incidence of *Wolbachia* in family Solanaceae was 33.33% in brinjal, absent in tomato and 10% in potato; whereas, in family Fabaceae it was 3.33% in soyabean, 3.33% in mungbean and 16.66% in subabool.

Single factor ANOVA was used to find the disparity of the endosymbiont infection in selected host populations. Among the family Solanaceae and Fabaceae it was noticed that there is no any significant variation found in the distribution frequency of *Arsenophonus* and *Wolbachia*; Whereas, a significant variation was observed in the distribution frequency of *Cardinium* and *Rickettsia* with the p-values as 0.0003 and 0.001 respectively.

### Phylogenetic analysis

**Figure 2.**
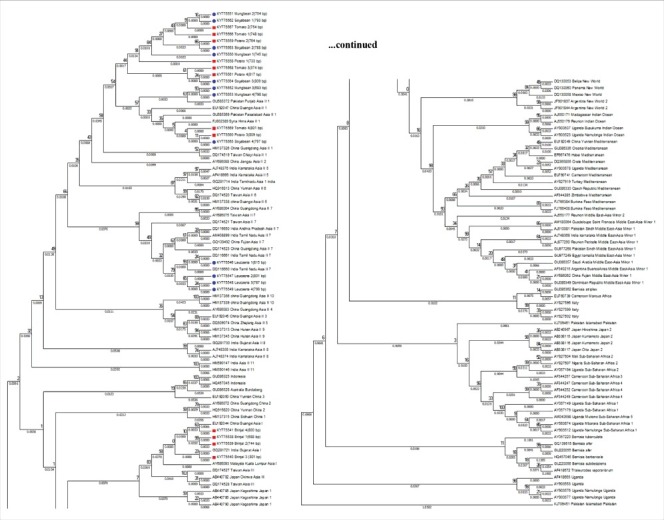
Phylogram of the evaluated *Bemisia tabaci* samples with well-assigned homologous sequences of the *B*. *tabaci* genetic groups from the consensus sequence database by means of maximum likelihood (ML) tree method and the kimura 2-parameter distances mitochondrial COI sequences.

**Figure 3.**
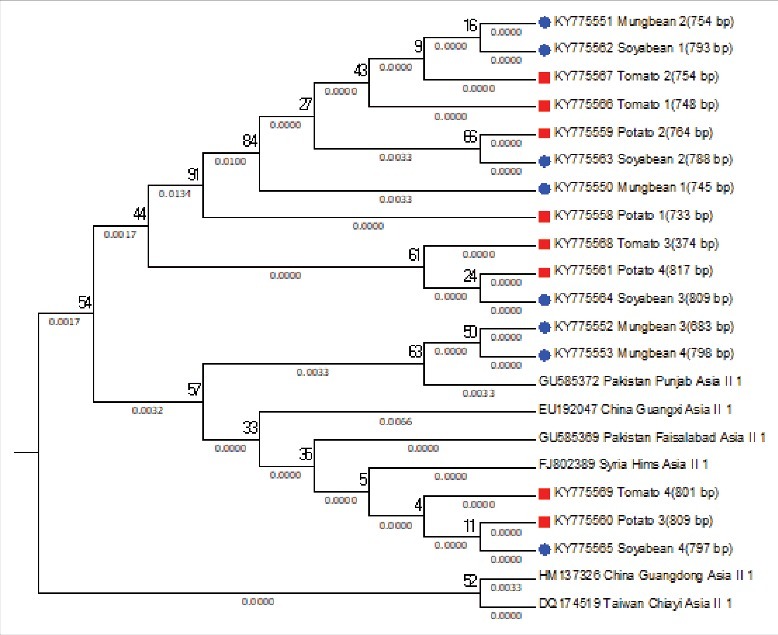
Subtree of evaluated *Bemisia tabaci* samples aligning with Asia II 1 genetic group with well-assigned homologous sequences from the consensus sequence database by means of maximum likelihood (ML) tree method and the kimura 2-parameter distances mitochondrial COI sequences.

**Figure 4.**
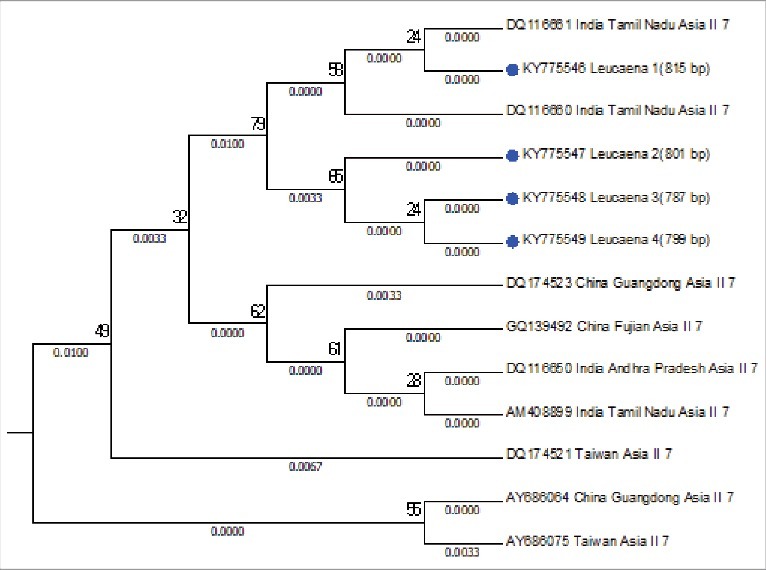
Subtree of evaluated *Bemisia tabaci* samples aligning with Asia II 7 genetic group with well-assigned homologous sequences from the consensus sequence database by means of maximum likelihood (ML) tree method and the kimura 2-parameter distances mitochondrial COI sequences.

**Figure 5.**
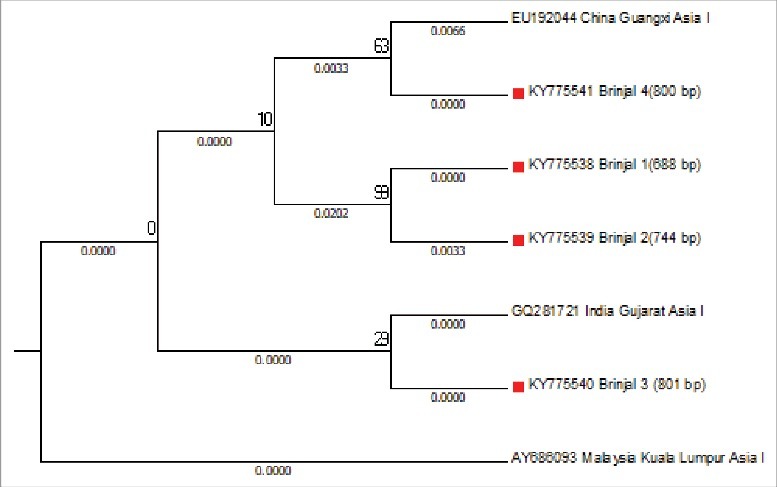
Subtree of evaluated *Bemisia tabaci* samples aligning with Asia 1 genetic group with well-assigned homologous sequences from the consensus sequence database by means of maximum likelihood (ML) tree method and the kimura 2-parameter distances mitochondrial COI sequences.

The mtCOI sequences of *B. tabaci* were studied for the determination of genetic group. Family Solanaceae and Fabaceae were showed the presence of diverse range of genetic groups, as the samples from the families aligned to Asia 1, Asia II 1 and Asia II 7 genetic groups (Fig. 2). Samples from tomato, potato, soyabean and mungbean were aligned to Asia II-1 genetic group (Fig. 3), samples collected from subabool was aligned to Asia II-7 genetic group (Fig. 4) and the samples from brinjal were settled down with the Asia 1 genetic group (Fig. 5).

## Discussion

The *B. tabaci* individuals collected from solanaceous and fabaceous hosts were settled with Asia 1, Asia II 1 and Asia II 7 genetic group after phylogenetic analysis. From the solanaceous hosts brinjal individuals were aligned to Asia 1 genetic group; tomato and potato individuals were aligned to Asia II 1 genetic group. While from fabaceous hosts soyabean and mungbean individuals were aligned to Asia II 1 genetic group; subabool individuals were aligned to Asia II 7 genetic group. The results are in agreement with the conclusions of Ellango et al. (2015) [15], which states that the Asia II 1 and Asia II 7 are the notable genetic group in Delhi. His interpretation also established that out of 34 putative species only nine species have been recognized from India. They also suggested that maximum variety is present in southern and eastern India.

An investigation of individuals from solanaceous and fabaceous hosts revealed a divergence in the distribution frequency of secondary endosymbionts with host preferences and genetic groups. This study recommends the relation among the symbiotic bacterial populations and the genetic groups of *B. tabaci* as prior demarcated by the researchers [28,29,39]. The consequence found between whitefly biotypes and secondary symbionts proposes a believable result of these bacteria to host features such as insecticide resistance, host range, virus transmission and speciation [28].

Insects are identified for having endosymbiotic microorganisms producing a diversity of symbiotic relations stretching from mutualism to parasitism [40,41]. The mutualistic link of symbiotic bacteria express a noteworthy appeal by providing crucial nutrients which are absent in arthropods because of nourishing on imbalanced foods such as plant sap [42]. Besides providing nutrients, these bacterial endosymbionts are moreover documented to have a range of effect on their hosts, *viz.,* increasing the range of temperature tolerance [43], enhanced resistance to parasites [21] and possibly have a role in the sexual assortment of its insect hosts [44].

The percentage distribution of *Cardinium* was higher in individuals belonging to Asia II 1 genetic group. As the results describes the percentage presence of *Cardinium* in tomato, potato, soyabean and mungbean individuals were 100%, 93.33%, 76.67% and 96.67% respectively. The lowest presence of *Cardinium* was noted in Asia II 7 genetic group (subabool, 13.34%), followed by Asia 1 genetic group (brinjal, 20%). For *Rickettsia* percentage presence was higher in Asia 1 genetic group (brinjal, 90%), whereas an average percentage distribution was recorded in Asia II 1 and Asia II 7 genetic groups (Fig. 1). In case of *Wolbachia*, again the presence was higher in Asia 1 genetic group (brinjal, 33.33%), whereas in Asia II 1 genetic group it was found in least proportion as the potato harbors 10%, soyabean and mungbean harbors 3.33% and interestingly it was totally absent in tomato individuals. The percentage distribution of *Wolbachia* was 16.66% in Asia II 7 genetic group (subabool). The distribution frequency of *Arsenophonus* was recorded higher in subabool individuals as 90% belonging to Asia II 7 genetic group. Whereas, the percentage presence of *Arsenophonus* was least recorded in the brinjal individuals as 16.66% belonging to Asia 1 genetic group. These outcomes are in contract with the earlier reviews by Chiel et al. (2007), Gueguen et al. (2010) and Gnankine´ et al. (2013) [28,29,39] which enumerated that inside Q genetic group, most Q 1 individual's harbour *Hamiltonella* and rarely low frequencies of *Wolbachia* and *Cardinium*, while Q 3 individuals harbour commonly *Arsenophonus* with a high level of co-infection with *Rickettsia*. In added words, there is an inequality in the distribution of secondary endosymbionts inside varied genetic groups of *B. tabaci*.

The study is trying to document the distribution incidence the secondary endosymbionts related with host's preference of *B. tabaci* in New Delhi, India. The results point out an inevitability for groundbreaking revisions on the host wise occurrence of secondary endosymbionts and its link with different genetic groups of *B. tabaci* and their impact in the polyphagous nature of this insect pest.

## Material and methods

### Sample collection and DNA extraction

*Bemisia tabaci* samples were collected from fields of the Indian Agricultural Research Institute, New Delhi, during 2016 and 2017, and preserved particularly in Eppendorf tubes with absolute alcohol at -20^0^C until further handling. Sample size of 180 individuals from six hosts of two families were used for the study. Whole genomic DNA of each adult single fly was retrieve by means of DNASure Tissue Mini Kit (Nucleo- pore, Genetix) as per manufacturer's protocol and kept at -20°C. The extracted DNA was used in the succeeding experiments.

### Identification of genetic group

The identification of the genetic group of *B. tabaci* was concluded aground on mitochondrial cytochrome oxidase I (mtCOI), subsequently a PCR reaction with universal primers [35] (Table 1). The finishing size of the PCR mixture was 25 µl comprising of 12.5 µl of Thermo Scientific maxima hot start PCR master mix, 8.5 µl of molecular grade water, 1 µl each forward primer CI-J-2195 and reverse primer TL2-N-3014 and 2 µl of genomic DNA. Ventri® 96- well thermal cycler (Applied Biosystems® Life Technologies) was used for the extension of the samples. PCR program used for the amplification of mtCO1 region is presented in Table 2. The amplified products were determined in 1% agarose gel, stained by ethidium bromide and envisaged in a gel documentation system (DNr, Bio-Imaging systems, MiniLumi). By the estimated band (Table 1) size of the gels, the products were used for sequencing.

**Table 2.** PCR programs used to detect the prevalence of primary and secondary endosymbionts in *Bemisia tabaci*.

			Cycling conditions		
Endosymbionts	Pre- denaturation	Denaturation	Annealing	Extension	Cycles
*Portiera*	94°C (4 Min)	94°C (30 s)	56°C (2 Min)	72°C (2 Min)	35
*Wolbachia*	94°C (4 Min)	94°C (30 s)	55°C (2 Min)	72°C (2 Min)	35
*Arsenophonus*	94°C (4 Min)	94°C (30 s)	56°C (2 Min)	72°C (2 Min)	35
*Cardinium*	94°C (4 Min)	94°C (30 s)	52°C (2 Min)	72°C (2 Min)	35
*Rickettsia*	94°C (4 Min)	94°C (30 s)	58°C (2 Min)	72°C (2 Min)	35
*B. tabaci*	94°C (1 Min)	94°C (1 Min)	55°C (1 Min)	72°C (1 Min)	35

Genetic group identification was done by sequence evaluations using the web-based Basic Local Alignment Search Tool algorithm of NCBI (https://blast.ncbi.nlm.nih.gov/Blast.cgi). The genetic group uniqueness was additionally recognized by the phylogenetic and molecular evolutionary analysis with well-assigned homologous sequences of the *B. tabaci* genetic groups from the consent sequence databank by MEGA version 6 [9,36]. The mtCOI sequences in FASTA format were bring together into the sequence alignment application of MEGA 6^36^ and multiple sequence alignments were attained with the Clustal W^37^ algorithm using default parameters. The sequences were submitted to NCBI for GenBank Accessions (Table 2). Sequence divergences among *B. tabaci* samples were assessed using the Kimura 2-Parameter distance model [38] and graphically revealed in a maximum likelihood (ML) tree by the program MEGA 6 [36]. Tree robustness was assessed by bootstrapping with 2,000 replicates with the *Bemisia afer*, *Bemisia atriplex*, *Bemisia berbericola*, *Bemisia subdecipiens*, *Bemisia tuberculate* and *Trialeurodes vaporariorum* as outgroups.

### Screening of endosymbionts

Genus specific primers amplifying the 16S rRNA gene for *Portiera, Rickettsia*, *Wolbachia* and *Cardinium* and the 23S rRNA gene for *Arsenophonus* (Table 1) were used for the authorization of endosymbionts occurrence in the individuals from each host belonging to different families. The PCR programs for the extension of bacterial endosymbionts are shown in Table 3. The products were envisioned in 1.0% agarose gel comprising ethidium bromide. Through the expected band size (Table 1) on the gel, products were used for sequencing. The acquired sequences were equated with the sequences on GenBank via BLAST algorithm in NCBI. For each endosymbiont, arbitrarily five samples were sequenced for reference band size and submitted to NCBI for GenBank Accessions (Table 4).

**Table 3.** Showing GenBank Accession numbers, along with locality data of mtCOI.

S.No.	Species	Genetic group	Location	GenBank Sequence ID
1.	*Bemisia tabaci*	Asia 1	India: Tamilnadu	GQ281714
2.	*Bemisia tabaci*	Asia 1	China: Guangxi	EU192044
3.	*Bemisia tabaci*	Asia 1	Malaysia: Kuala Lumpur	AY686093
4.	*Bemisia tabaci*	Asia 1	India: Gujarat	GQ281721
5.	*Bemisia tabaci*	Asia II 2	China: Jiangsu	AY686088
6.	*Bemisia tabaci*	Asia II 3	China: Guangxi	EU192045
7.	*Bemisia tabaci*	Asia II 3	China: Zhejiang	DQ309074
8.	*Bemisia tabaci*	Asia II 4	China: Guangdong	AY686083
9.	*Bemisia tabaci*	Asia II 5	India: Karnataka	AJ748376
10.	*Bemisia tabaci*	Asia II 5	India: Karnataka	AF418666
11.	*Bemisia tabaci*	Asia II 6	Taiwan	DQ174520
12.	*Bemisia tabaci*	Asia II 6	China: Guangxi	HM137338
13.	*Bemisia tabaci*	Asia II 6	China: Yunnan	HQ916813
14.	*Bemisia tabaci*	Asia II 8	India: Karnataka	AJ748358
15.	*Bemisia tabaci*	Asia II 8	India: Karnataka	AJ748374
16.	*Bemisia tabaci*	Asia II 8	India: Gujarat	GQ281733
17.	*Bemisia tabaci*	Asia II 9	China: Hunan	HM137313
18.	*Bemisia tabaci*	Asia II 9	China: Hunan	HM137345
19.	*Bemisia tabaci*	Asia II 10	China: Guangdong	HM137356
20.	*Bemisia tabaci*	Asia II 10	China: Guangdong	HM137339
21.	*Bemisia tabaci*	Asia II 1	China: Guangdong	HM137326
22.	*Bemisia tabaci*	Asia II 1	China: Guangxi	EU192047
23.	*Bemisia tabaci*	Asia II 1	Pakistan: Faisalabad	GU585369
24.	*Bemisia tabaci*	Asia II 1	Syria: Hims	FJ802389
25.	*Bemisia tabaci*	Asia II 1	Taiwan: Chiayi	DQ174519
26.	*Bemisia tabaci*	Asia II 1	Pakistan: Punjab	GU585372
27.	*Bemisia tabaci*	Asia II 7	China: Guangdong	AY686064
28.	*Bemisia tabaci*	Asia II 7	Taiwan	AY686075
29.	*Bemisia tabaci*	Asia II 7	India: Tamil Nadu	DQ116660
30.	*Bemisia tabaci*	Asia II 7	Taiwan	DQ174521
31.	*Bemisia tabaci*	Asia II 7	China: Guangdong	DQ174523
32.	*Bemisia tabaci*	Asia II 7	China: Fujian	GQ139492
33.	*Bemisia tabaci*	Asia II 7	India: Andhra Pradesh	DQ116650
34.	*Bemisia tabaci*	Asia II 7	India: Tamil Nadu	DQ116661
35.	*Bemisia tabaci*	Asia II 7	India: Tamil Nadu	AM408899
36.	*Bemisia tabaci*	Asia III	Japan: Okinwa	AB440792
37.	*Bemisia tabaci*	Asia III	Taiwan	DQ174527
38.	*Bemisia tabaci*	Asia III	Taiwan	DQ174528
39.	*Bemisia tabaci*	Australia	Australia	GU086328
40.	*Bemisia tabaci*	Indonesia	Indonesia	GU086325
41.	*Bemisia tabaci*	Indonesia	Indonesia	HQ457045
42.	*Bemisia tabaci*	China 1	China: Sichuan	HM137315
43.	*Bemisia tabaci*	China 2	China: Guangdong	AY686072
44.	*Bemisia tabaci*	China 2	China: Yunnan	HQ916820
45.	*Bemisia tabaci*	China 3	China: Yunnan	EU192050
46.	*Bemisia tabaci*	Indian Ocean	Madagascar	AJ550171
47.	*Bemisia tabaci*	Indian Ocean	Reunion	AJ550179
48.	*Bemisia tabaci*	Indian Ocean	Uganda: Busukuma	AY903537
49.	*Bemisia tabaci*	Indian Ocean	Uganda: Namulonge	AY903523
50.	*Bemisia tabaci*	Italy	Italy	AY827596
51.	*Bemisia tabaci*	Italy	Italy	AY827599
52.	*Bemisia tabaci*	Italy	Italy	AY827602
53.	*Bemisia tabaci*	Japan 2	Japan: Hiroshima	AB240967
54.	*Bemisia tabaci*	Japan 2	Japan: Kumamoto	AB308115
55.	*Bemisia tabaci*	Japan 2	Japan: Kumamoto	AB308116
56.	*Bemisia tabaci*	Japan 2	Japan: Oita	AB308117
57.	*Bemisia tabaci*	Mediterranean	Burkina Faso	FJ766384
58.	*Bemisia tabaci*	Mediterranean	Burkina Faso	FJ766408
59.	*Bemisia tabaci*	Mediterranean	Cameroon	EU760741
60.	*Bemisia tabaci*	Mediterranean	Hubei	EF667476
61.	*Bemisia tabaci*	Mediterranean	China: Yunnan	EU192049
62.	*Bemisia tabaci*	Mediterranean	Crete	DQ365856
63.	*Bemisia tabaci*	Mediterranean	Croatia	GU086336
64.	*Bemisia tabaci*	Mediterranean	Czech Republic	GU086330
65.	*Bemisia tabaci*	Mediterranean	Turkey	AY827619
66.	*Bemisia tabaci*	Mediterranean	Uganda	AY903578
67.	*Bemisia tabaci*	Mediterranean	Zimbabwe	AF344285
68.	*Bemisia tabaci*	Middle East Asia Minor 1	Argentina: Buenos Aires	AF340215
69.	*Bemisia tabaci*	Middle East Asia Minor 1	China: Fujian	AY686062
70.	*Bemisia tabaci*	Middle East Asia Minor 1	Dominican Republic	GU086349
71.	*Bemisia tabaci*	Middle East Asia Minor 1	Egypt: Ismailia	GU977249
72.	*Bemisia tabaci*	Middle East Asia Minor 1	Guadeloupe: Saint Francois	AM180064
73.	*Bemisia tabaci*	Middle East Asia Minor 1	India: Karnataka	AJ748368
74.	*Bemisia tabaci*	Middle East Asia Minor 1	Pakistan: Sindh	AJ510081
75.	*Bemisia tabaci*	Middle East Asia Minor 1	Pakistan: Sindh	GU977268
76.	*Bemisia tabaci*	Middle East Asia Minor 1	Reunion: Petitelle	AJ877260
77.	*Bemisia tabaci*	Middle East Asia Minor 1	Saudi Arabia	GU086357
78.	*Bemisia tabaci*	Middle East Asia Minor 2	Reunion	AJ550177
79.	*Bemisia tabaci*	New World	Belize	DQ130053
80.	*Bemisia tabaci*	New World	Mexico	DQ130058
81.	*Bemisia tabaci*	New World	Panama	DQ130060
82.	*Bemisia tabaci*	New World 2	Argentina	JF901837
83.	*Bemisia tabaci*	New World 2	Argentina	JF901844
84.	*Bemisia afer*		Outgroup	GQ139515
85.	*Bemisia afer*		Outgroup	GU220055
86.	*Bemisia atriplex*		Outgroup	GU086362
87.	*Bemisia berbericola*		Outgroup	HQ457046
88.	*Bemisia subdecipiens*		Outgroup	GU220056
89.	*Bemisia tuberculate*		Outgroup	AY057220
90.	*Trialeurodes vaporariorum*		Outgroup	AF418672
91.	*Bemisia tabaci*	Sub-Saharan Africa 1	Uganda	AY057149
92.	*Bemisia tabaci*	Sub-Saharan Africa 1	Uganda: Mbarara	AY563674
93.	*Bemisia tabaci*	Sub-Saharan Africa 1	Uganda: Namulonge	AY903512
94.	*Bemisia tabaci*	Sub-Saharan Africa 1	Uganda	AY057179
95.	*Bemisia tabaci*	Sub-Saharan Africa 2	Uganda	AY057194
96.	*Bemisia tabaci*	Sub-Saharan Africa 2	Mali	AY827604
97.	*Bemisia tabaci*	Sub-Saharan Africa 2	Nigeria	AY827607
98.	*Bemisia tabaci*	Sub-Saharan Africa 2	Spain	GU086361
99.	*Bemisia tabaci*	Sub-Saharan Africa 3	Cameroon	AF344257
100.	*Bemisia tabaci*	Sub-Saharan Africa 4	Cameroon	AF344247
101.	*Bemisia tabaci*	Sub-Saharan Africa 4	Cameroon	AF344249
102.	*Bemisia tabaci*	Sub-Saharan Africa 4	Cameroon	AF344252
103.	*Bemisia tabaci*	Uganda	Uganda	AY903553
104.	*Bemisia tabaci*	Uganda	Uganda: Namulonge	AY903576
105.	*Bemisia tabaci*	Uganda	Uganda: Namulonge	AY903577
106.	*Bemisia tabaci*	Uganda	Uganda	AF418665
107.	*Bemisia tabaci*	Asia II 11	India	HM590147
108.	*Bemisia tabaci*	Asia II 11	India	HM590146
109.	*Bemisia tabaci*	Africa	Cameroon: Maroua	EU760739
110.	*Bemisia tabaci*	Sub-Saharan Africa 5	Uganda: Mukono	AM040598
111.	*Bemisia tabaci*	Japan 1	Japan: Kagoshima	AB440785
112.	*Bemisia tabaci*	Japan 1	Japan: Kagoshima	AB440786
113.	*Bemisia tabaci*	Japan 1	Japan: Kagoshima	AB440790
114.	*Bemisia tabaci*	Pakistan	Pakistan: Islamabad	KJ709461
115.	*Bemisia tabaci*	Pakistan	Pakistan: Islamabad	KJ709451
116.	*Bemisia tabaci*	Asia II 7	India: Delhi	KY775546^*^
117.	*Bemisia tabaci*	Asia II 7	India: Delhi	KY775547^*^
118.	*Bemisia tabaci*	Asia II 7	India: Delhi	KY775548^*^
119.	*Bemisia tabaci*	Asia II 7	India: Delhi	KY775549^*^
120.	*Bemisia tabaci*	Asia II 1	India: Delhi	KY775562^*^
121.	*Bemisia tabaci*	Asia II 1	India: Delhi	KY775563^*^
122.	*Bemisia tabaci*	Asia II 1	India: Delhi	KY775564^*^
123.	*Bemisia tabaci*	Asia II 1	India: Delhi	KY775565^*^
124.	*Bemisia tabaci*	Asia II 1	India: Delhi	KY775550^*^
125.	*Bemisia tabaci*	Asia II 1	India: Delhi	KY775551^*^
126.	*Bemisia tabaci*	Asia II 1	India: Delhi	KY775552^*^
127.	*Bemisia tabaci*	Asia II 1	India: Delhi	KY775553^*^
128.	*Bemisia tabaci*	Asia II 1	India: Delhi	KY775558^*^
129.	*Bemisia tabaci*	Asia II 1	India: Delhi	KY775559^*^
130.	*Bemisia tabaci*	Asia II 1	India: Delhi	KY775560^*^
131.	*Bemisia tabaci*	Asia II 1	India: Delhi	KY775561^*^
132.	*Bemisia tabaci*	Asia II 1	India: Delhi	KY775566^*^
133.	*Bemisia tabaci*	Asia II 1	India: Delhi	KY775567^*^
134.	*Bemisia tabaci*	Asia II 1	India: Delhi	KY775568^*^
135.	*Bemisia tabaci*	Asia II 1	India: Delhi	KY775569^*^
136.	*Bemisia tabaci*	Asia 1	India: Delhi	KY775538^*^
137.	*Bemisia tabaci*	Asia 1	India: Delhi	KY775539^*^
138.	*Bemisia tabaci*	Asia 1	India: Delhi	KY775540^*^
139.	*Bemisia tabaci*	Asia 1	India: Delhi	KY775541^*^

* Sequences obtained from the current study.

**Table 4.** Showing the GenBank Accession number of submitted reference sequences of endosymbionts.

S.No.	Gene	Organism	GenBank Accession Number
1.	16S rRNA	*Portiera*	KX161849
2.	16S rRNA	*Portiera*	KX161850
3.	16S rRNA	*Portiera*	KX161851
4.	16S rRNA	*Portiera*	KX161852
5.	16S rRNA	*Portiera*	KX161853
6.	16S rRNA	*Cardinium*	KX197216
7.	16S rRNA	*Cardinium*	KX197217
8.	16S rRNA	*Cardinium*	KX197218
9.	16S rRNA	*Cardinium*	KX197219
10.	16S rRNA	*Cardinium*	KX197220
11.	16S rRNA	*Rickettsia*	KX197221
12.	16S rRNA	*Rickettsia*	KX197222
13.	16S rRNA	*Rickettsia*	KX197223
14.	16S rRNA	*Rickettsia*	KX197224
15.	16S rRNA	*Rickettsia*	KX197225
16.	16S rRNA	*Wolbachia*	KX197226
17.	16S rRNA	*Wolbachia*	KX197227
18.	16S rRNA	*Wolbachia*	KX197228
19.	16S rRNA	*Wolbachia*	KX197229
20.	16S rRNA	*Wolbachia*	KX197230
21.	23S rRNA	*Arsenophonus*	KY630534
22.	23S rRNA	*Arsenophonus*	KY630535
23.	23S rRNA	*Arsenophonus*	KY630536
24.	23S rRNA	*Arsenophonus*	KY630537

The divergences in comparative number of endosymbionts in *B. tabaci* were examined by means of one-way analysis of variance (ANOVA). Statistical assessments were completed with SPSS version 16.0.

## Conclusion

The present study was concentrated on finding the endosymbiont array associated with *B. tabaci* on Solanaceae and Fabaceae host plants of New Delhi, India. The consequences originated an agreement that there is a breach in the facts of existence of secondary endosymbionts with respect to the host plants and genetic groups; and suggests an obligation for lenient enhancements on the host wise occurrence of secondary endosymbionts and its stretch with numerous genetic groups. A broadminded and proportionate survey is obligatory to reveal the confirmations concerning the role of these endosymbionts and the source of uneven passage frequency of these secondary endosymbionts.
